# The Antimicrobial Peptide lin-SB056-1 and Its Dendrimeric Derivative Prevent *Pseudomonas aeruginosa* Biofilm Formation in Physiologically Relevant Models of Chronic Infections

**DOI:** 10.3389/fmicb.2019.00198

**Published:** 2019-02-08

**Authors:** Lucia Grassi, Giovanna Batoni, Lisa Ostyn, Petra Rigole, Sara Van den Bossche, Andrea C. Rinaldi, Giuseppantonio Maisetta, Semih Esin, Tom Coenye, Aurélie Crabbé

**Affiliations:** ^1^Department of Translational Research and New Technologies in Medicine and Surgery, University of Pisa, Pisa, Italy; ^2^Laboratory of Pharmaceutical Microbiology, Ghent University, Ghent, Belgium; ^3^Department of Biomedical Sciences, University of Cagliari, Cagliari, Italy

**Keywords:** antimicrobial peptides, dendrimeric peptide, combination treatment, biofilm, *Pseudomonas aeruginosa*, 3-D lung epithelial cell model, artificial wound model

## Abstract

Antimicrobial peptides (AMPs) are promising templates for the development of novel antibiofilm drugs. Despite the large number of studies on screening and optimization of AMPs, only a few of these evaluated the antibiofilm activity in physiologically relevant model systems. Potent *in vitro* activity of AMPs often does not translate into *in vivo* effectiveness due to the interference of the host microenvironment with peptide stability/availability. Hence, mimicking the complex environment found in biofilm-associated infections is essential to predict the clinical potential of novel AMP-based antimicrobials. In the present study, we examined the antibiofilm activity of the semi-synthetic peptide lin-SB056-1 and its dendrimeric derivative (lin-SB056-1)_2_-K against *Pseudomonas aeruginosa* in an *in vivo*-like three-dimensional (3-D) lung epithelial cell model and an *in vitro* wound model (consisting of an artificial dermis and blood components at physiological levels). Although moderately active when tested alone, lin-SB056-1 was effective in reducing *P. aeruginosa* biofilm formation in association with 3-D lung epithelial cells in combination with the chelating agent EDTA. The dimeric derivative (lin-SB056-1)_2_-K demonstrated an enhanced biofilm-inhibitory activity as compared to both lin-SB056-1 and the lin-SB056-1/EDTA combination, reducing the number of biofilm-associated bacteria up to 3-Log units at concentrations causing less than 20% cell death. Biofilm inhibition by (lin-SB056-1)_2_-K was reported both for the reference strain PAO1 and cystic fibrosis lung isolates of *P. aeruginosa*. In addition, using fluorescence microscopy, a significant decrease in biofilm-like structures associated with 3-D cells was observed after peptide exposure. Interestingly, effectiveness of (lin-SB056-1)_2_-K was also demonstrated in the wound model with a reduction of up to 1-Log unit in biofilm formation by *P. aeruginosa* PAO1 and wound isolates. Overall, combination treatment and peptide dendrimerization emerged as promising strategies to improve the efficacy of AMPs, especially under challenging host-mimicking conditions. Furthermore, the results of the present study underlined the importance of evaluating the biological properties of novel AMPs in *in vivo*-like model systems representative of specific infectious sites in order to make a more realistic prediction of their therapeutic success, and avoid the inclusion of unpromising peptides in animal studies and clinical trials.

## Introduction

The opportunistic pathogen *Pseudomonas aeruginosa* is known to play a role in many types of infections, including lung infections, wound infections, otitis media and medical device-associated infections (Taylor et al., 2014; Rybtke et al., 2015). *P. aeruginosa* infections range from minor external infections to serious and life-threatening conditions, such as in individuals with CF (Gellatly and Hancock, 2013). Intrinsic and acquired resistance to many conventional antibiotics makes the treatment of *P. aeruginosa* infections particularly challenging (Breidenstein et al., 2011). The ability of *P. aeruginosa* to form biofilms further complicates the problem of antimicrobial resistance causing high rates of failure in antibiotic therapy and chronic infection of the host (Parsek and Singh, 2003; Mulcahy et al., 2014). Therefore, the identification of novel antimicrobial agents that can effectively suppress *P. aeruginosa* biofilm-related infections is a research priority.

Over the last years, AMPs have gained increasing attention as candidates for the development of innovative antimicrobial drugs. Due to their main mechanism of action, which relies on the permeabilization of bacterial membranes, AMPs exhibit broad-spectrum activity, a low tendency to induce resistance, and a high potential to target metabolically dormant cells that are found at elevated rate within microbial biofilms (Batoni et al., 2016b; Grassi et al., 2017a). A number of AMPs have been tested for their antibiofilm activity (BaAMPs database^1^), demonstrating a variable effectiveness in interfering with the different stages of biofilm formation (Di Luca et al., 2015; de la Fuente-Núñez et al., 2016). Despite the large number of studies reporting the screening and optimization of AMPs, only a few of these evaluated the antibiofilm activity in clinically relevant model systems (Lashua et al., 2016; Crabbé et al., 2017; de Breij et al., 2018). Nevertheless, the specific environmental conditions found at the site of infection have been shown to influence both biofilm architecture and susceptibility and to interfere with the activity and stability of AMPs (Bjarnsholt et al., 2013; Crabbé et al., 2017). For this reason, potent *in vitro* activity of AMPs does not necessarily translate into *in vivo* effectiveness, resulting in a limited predictive value (Mardirossian et al., 2017; Dostert et al., 2018). Therefore, the screening of AMPs in conditions mimicking the host microenvironment and the *in vivo* host–pathogen interactions represents an essential step to assess the clinical potential of novel AMP-based antimicrobials.

The semi-synthetic peptide lin-SB056-1 has been recently designed as a beta-strand with a perfect pattern of alternating hydrophilic and hydrophobic residues in the primary structure in order to maximize bacterial membrane interaction and killing (Manzo et al., 2015). The regular amphipathic profile of lin-SB056-1 significantly enhanced the antibacterial properties of the peptide, which displayed a rapid and strong activity against a broad spectrum of Gram-positive and Gram-negative bacteria, including multidrug-resistant clinical isolates of *P. aeruginosa* (Maisetta et al., 2017). In addition to exerting a marked antibiofilm activity in standard conditions, lin-SB056-1 in combination with EDTA demonstrated the ability to almost completely inhibit the formation of *P. aeruginosa* biofilm-like structures in an artificial sputum medium mimicking the lung environment of CF patients (Maisetta et al., 2017). Since dendrimeric peptides are known to exhibit enhanced antimicrobial activity as compared to their monomeric counterparts (Lind et al., 2015; Pires et al., 2015), lin-SB056-1 has been further optimized by generating a two-branched polypeptide with a lysine linker and a lipidic tail (den-SB056-1) (Batoni et al., 2016a). Interestingly and differently from the monomeric structure, den-SB056-1 has been demonstrated to preserve high affinity for negatively charged membranes and to retain its antibacterial activity even in the presence of physiological salt concentrations (Batoni et al., 2016a). Since the inhibition of AMPs by salts represents a severe limitation to their applicability (Maisetta et al., 2008), maintenance of activity at high ionic strength makes the dendrimeric derivative particularly promising for the use in physiological conditions (Polcyn et al., 2013). In the current study, a slightly different dimeric derivative of lin-SB056-1, referred to as (lin-SB056-1)_2_-K, has been obtained starting from den-SB056-1 through the elimination of the lipidic moiety. Indeed, a recent study has reported that the lipidic tail does not play a fundamental role in the membrane-binding affinity as well as in the antimicrobial properties of the peptide (Manzo et al., 2016).

In the present study, combination treatment (i.e., lin-SB056-1 used in combination with EDTA) and peptide dendrimerization [i.e., design of (lin-SB056-1)_2_-K] were investigated as two promising strategies to improve the antibacterial and antibiofilm activity of lin-SB056-1 against *P. aeruginosa* under *in vivo*-like conditions. In particular, the antimicrobial properties of the lin-SB056-1/EDTA combination and/or (lin-SB056-1)_2_-K were assessed in two representative model systems of biofilm-associated infections, namely an *in vivo*-like three-dimensional (3-D) lung epithelial cell model and an artificial wound model. Organotypic 3-D lung models have been previously developed by using the RWV bioreactor technology, which allows to replicate aspects of the *in vivo* lung infection with *P. aeruginosa* biofilms (Carterson et al., 2005; Barrila et al., 2010; Crabbé et al., 2014, 2017). In order to mimic chronic wound infections, we exploited an *in vitro* wound model reflecting the nutritional conditions found in wound beds, and resembling the actual surface on which bacterial aggregates are formed *in vivo* (Brackman and Coenye, 2016; Trivedi et al., 2017). Overall, the results demonstrated the ability of the lin-SB056-1/EDTA combination to retain its biofilm-inhibitory activity in the presence of the host cellular component that is known to markedly influence the effectiveness of both antibiotics and AMPs (Moreau-Marquis et al., 2008; Crabbé et al., 2017). Furthermore, the dendrimeric peptide (lin-SB056-1)_2_-K displayed an enhanced ability in inhibiting *P. aeruginosa* biofilm formation as compared to both lin-SB056-1 and the lin-SB056-1/EDTA combination, thereby underlining the importance of the rational peptide optimization to obtain valid templates to be developed as novel antibiofilm agents. Importantly, the present study highlighted the need of including physiologically relevant model systems in the preclinical testing of candidate antibiofilm peptides in order to assess their potential, but also their limitations.

## Materials and Methods

### AMPs and EDTA

Lin-SB056-1 (KWKIRVRLSA-NH_2_) and (lin-SB056-1)_2_-K ([KWKIRVRLSA]_2_-K) were synthetized by Peptide Protein Research (Fareham, England). Analysis of the synthetic peptides by high performance liquid chromatography and mass spectrometry revealed purity over 98%. Peptides were diluted in milli-Q water to obtain a stock solution of 2 mg/mL and stored at -80°C. Disodium EDTA was purchased from Sigma-Aldrich (St. Louis, MO, United States). A stock solution of EDTA (50 mM, pH 8) was prepared in milli-Q water, sterilized by filtration and stored at 4°C.

### Bacterial Strains and Culture Conditions

The reference strain *P. aeruginosa* PAO1 (ATCC 15692) was used in this study as well as clinical isolates of *P. aeruginosa* (AA2 and AA44) from the lungs of patients with CF (Cullen et al., 2015). *P. aeruginosa* burn wound isolates (2091 and 2549) were obtained from Ghent University Hospital (Ghent, Belgium) (Zhang et al., 2018). For experiments involving fluorescence imaging of host-associated biofilms, the previously reported GFP-expressing strain of *P. aeruginosa* PAO1 was used (Crabbé et al., 2017). All strains were stored in a cryovial bead storage system (Microbank, Pro-Lab Diagnostics, Metropolitan Borough of Wirral, United Kingdom) at -80°C. For the preparation of the inoculum, beads from the frozen cultures were spread on TSA (LabM) and incubated overnight at 37°C. Liquid cultures were prepared starting from colonies isolated on agar plates. For all studies, *P. aeruginosa* PAO1, GFP-tagged *P. aeruginosa* PAO1 and *P. aeruginosa* clinical isolates from CF patients (AA2 and AA44) were aerobically cultured in LB at 37°C, 250 rpm. *P. aeruginosa* clinical isolates from wounds (2091 and 2549) were aerobically grown in TSB (LabM) at 37°C.

### Determination of Minimal Inhibitory Concentration (MIC)

The susceptibility of *P. aeruginosa* PAO1 and CF clinical isolates to lin-SB056-1 and (lin-SB056-1)_2_-K was assessed in terms of MIC values according to the standard microdilution method (Clinical and Laboratory Standards Institute [CLSI], 2018). Bacteria were grown in Mueller-Hinton Broth (MHB, Oxoid, Basingstoke, United Kingdom) until exponential phase and diluted in the same medium to reach a final density of 1 × 10^7^ CFU/mL. A volume of 10 μL of the bacterial suspensions was added to 90 μL of MHB in the absence (viability control) or in the presence of the peptides at different concentrations (1.2–77 μM). MIC values of both peptides were also determined in the culture medium used to grow 3-D lung epithelial cells (i.e., GTSF-2 medium, as described in Section “Biofilm Model Systems”). MIC values were defined as the lowest concentration of each peptide resulting in the complete inhibition of visible growth after 24 h of incubation at 37°C.

### Bactericidal Activity in GTSF-2 Medium

Bactericidal activity of lin-SB056-1, used alone and in combination with EDTA, was evaluated against *P. aeruginosa* PAO1 in GTSF-2 medium, as described previously (Maisetta et al., 2017). Bacteria were grown in LB until exponential phase and diluted in GTSF-2 medium to reach a final density of 1 × 10^7^ CFU/mL. A volume of 10 μL of the bacterial suspensions was added to 90 μL of GTSF-2 medium containing lin-SB056-1 (19.25 and 38.5 μM), EDTA (0.3–1.25 mM) and different peptide/EDTA combinations. Bacteria suspended in GTSF-2 medium were used as viability control. Samples were incubated statically for 1.5 h (37°C, 5% CO_2_), subsequently diluted 10-fold in a PSS (0.9% NaCl) and plated on TSA for the CFU count.

### Biofilm Model Systems

A 3-D lung epithelial cell model and an artificial wound model were exploited to assess the biofilm-inhibitory activity of the AMPs under investigation. The 3-D lung model was generated by growing the human adenocarcinoma alveolar epithelial cell line A549 (ATCC CCL-185) on microcarrier beads in RWV bioreactors (Synthecon, Houston, TX, United States), as previously described (Carterson et al., 2005; Crabbé et al., 2017). 3-D cultures were generated in GTSF-2 medium (GE Healthcare, Diegem, Belgium) supplemented with 10% heat-inactivated FBS (Thermo Fisher Scientific, Waltham, MA, United States) and 1% penicillin/streptomycin (Thermo Fisher Scientific) and were used for infection studies after 11–14 days of culture in the RWV (37°C, 5% CO_2_). Immediately prior to use, 3-D A549 cells were washed once with HBSS and resuspended in GTSF-2 medium (without FBS and antibiotics) at a final density of 1 × 10^6^ cells/mL.

The artificial wound model included a dermis-like scaffold and blood components at physiological concentrations. A two-layered artificial dermis composed of HA (Lifecore Biomedical, Chaska, MN, United States) and collagen (type-I collagen from rat tail) (Col; Sigma-Aldrich) with dimensions of approximately 1 cm^3^ was prepared as previously reported (Brackman et al., 2016). The upper layer of the artificial dermis consisted of chemically cross-linked high-molecular-weight HA (HMW-HA), while the lower layer was prepared from a mixed solution of HMW-HA, low-molecular-weight HA (LMW-HA) and heat-denatured Col. The biphasic spongy sheet was obtained by lyophilisation, subsequently irradiated with a UV lamp to induce cross-linking between Col molecules and finally sterilized at 110°C for 1 h. At the time of use, artificial dermis was immersed into a WLM consisting of BB (LabM) supplemented with 50% heparinized bovine plasma (Sigma-Aldrich) and 5% freeze-thaw laked horse blood (Biotrading, Mijdrecht, Netherlands) (Brackman et al., 2016).

### Cytotoxicity in 3-D Lung Epithelial Cell Model

Cytotoxicity of the tested compounds toward 3-D A549 cells was assessed by measuring the enzymatic activity of cytosolic LDH released from dead cells. Upon maturation of the 3-D aggregates (11–14 days), a volume of 250 μL of the 3-D lung epithelial cells (containing 2.5 × 10^5^ cells) was transferred into flat-bottom 48-well plates. Lin-SB056-1 (9.6–77 μM), used alone and in combination with EDTA (0.3–1.25 mM), and (lin-SB056-1)_2_-K (9.6–77 μM) were added to the 3-D cells and incubated statically for 4 h at 37°C, 5% CO_2_. 3-D aggregates incubated in GTSF-2 medium alone were used as negative (cell viability) control, while cells lysed with 1% Triton-X100 served as positive (death) control. Following incubation, cell culture medium was collected and centrifuged at 2500 × *g* for 15 min in order to remove cell debris. The amount of LDH in 3-D cell supernatants was quantified using a LDH activity assay kit (Sigma-Aldrich) following manufacturer’s instructions. The cytotoxic effect was determined according to the following formula: Cytotoxicity (%) = [(LDH activity _tested compound_ – LDH activity _negative control_)/(LDH activity _positive control_ – LDH activity _negative control_)] × 100.

### Biofilm-Inhibitory Activity in 3-D Lung Epithelial Cell Model

The ability of lin-SB056-1, alone and in combination with EDTA, to inhibit biofilm formation in association with 3-D cells was evaluated against *P. aeruginosa* PAO1. Biofilm-inhibitory activity of (lin-SB056-1)_2_-K was assessed against both *P. aeruginosa* PAO1 and an isogenic pair of early and late clinical CF strains isolated from the same patient (AA2 and AA44) (Cullen et al., 2015). Bacteria were cultured overnight in LB (37°C, 250 rpm), suspended in GTSF-2 medium and added to 3-D A549 cells (2.5 × 10^5^ cells/well) into 48-well plates at a multiplicity of infection (MOI) of 15. Biofilm formation was determined in the absence (negative control) or presence of different concentrations of lin-SB056-1 (9.6–77 μM), used alone and in combination with EDTA (0.3 and 0.6 mM), and (lin-SB056-1)_2_-K (9.6–77 μM). After 4 h of incubation (37°C, 5% CO_2_), host-associated biofilm formation was quantified through plating and CFU counting as described previously (Crabbé et al., 2017). To this end, 3-D cells were transferred into new 48-well plates and rinsed twice with HBSS (Thermo Fisher Scientific) in order to remove non-attached bacteria. To assess bacterial adhesion to 3-D cells, biofilms were exposed to 0.1% Triton X-100, disrupted by vigorous pipetting, serially diluted in PSS and plated on TSA.

### Light and Fluorescence Microscopy

The effect of the tested compounds on morphology and integrity of 3-D A549 cells was determined through light microscopy using EVOS XL Core Imaging System (Life Technologies, Carlsbad, CA, United States). Qualitative evaluation of antibiofilm activity against GFP-tagged *P. aeruginosa* PAO1 in the 3-D lung epithelial cell model was performed by fluorescence microscopy using EVOS FL Auto Imaging System (Life Technologies). Light and fluorescence microscopy images were acquired with 20 and 40x objectives (total magnification of 599 and 1200x, respectively) and appropriate filter cubes. Imaging of 3-D aggregates was carried out following 4 h of incubation with the tested compounds both in the absence and presence of bacteria.

### Hemolysis Assay

Hemolytic activity of lin-SB056-1 and (lin-SB056-1)_2_-K was tested against hRBCs as previously described (Grassi et al., 2017c). Briefly, a suspension of hRBCs (5%, v/v) was mixed with various concentrations of the peptides (1.2 to 77 μM) into round-bottom polystyrene 96-well microplates. hRBCs suspended in PBS alone were used as negative control (0% hemolysis), while cells lysed with 0.1% (v/v) Triton X-100 were taken as positive control (100% hemolysis). After 1 h of incubation (37°C, static), microplates were centrifuged at 1000 × *g* for 10 min, 4°C. Supernatants were transferred into new plates and optical density at 450 nm (OD_450_) was measured using a microplate reader. The hemolytic activity was quantified according to the following formula: Hemolysis (%) = [(OD _peptide_ – OD _negative control_)/(OD _positive control_ – OD _negative control_)] × 100.

### Biofilm-Inhibitory Activity in Artificial Wound Model

The ability of (lin-SB056-1)_2_-K to inhibit biofilm formation in the artificial wound model was evaluated against *P. aeruginosa* PAO1 and two clinical isolates from wound infections. Bacteria were cultured overnight in TSB (37°C, static), washed and diluted in PSS to a final density of 1 × 10^6^ CFU/mL. Artificial dermis was placed into flat-bottom 24-well plates and soaked with 500 μL of WLM. A volume of 10 μL of the bacterial suspension was spotted on the top of each artificial dermis. Following inoculation, (lin-SB056-1)_2_-K at a final concentration of 19.25 μM (100 μL) was added to the artificial dermis. Finally, additional WLM (400 μL) was added around the artificial dermis in order to avoid dehydration and plates were incubated at 37°C for 16 h. Based on preliminary experiments, such incubation time was found to ensure adequate *P. aeruginosa* biofilm formation without significant degradation of the artificial dermis by bacterial secreted factors. After the incubation, the artificial dermis was rinsed once in PSS to remove non-adherent cells and placed in glass tubes containing 10 mL of PSS. Biofilm-forming bacteria were detached from the artificial dermis through three cycles of vortexing (30 s) and sonication (30 s; Branson 3510, Branson Ultrasonics Corporation, Danbury, CT, United States), serially diluted in PSS and pour-plated on TSA to determine the number of CFU.

### Statistical Analysis

All the experiments were performed at least in biological triplicate, unless otherwise specified. Statistical analysis was carried out using SPSS statistics software, version 25 (SPSS, Chicago, IL, United States). Normal distribution of the data was verified using the Shapiro–Wilk test. In the case of normally distributed data, differences between mean values were evaluated with the Student’s *t*-test for independent samples. Non-normally distributed data were analyzed using a Mann–Whitney test and Kruskal–Wallis test followed by Bonferroni *post hoc* test. In the case of combination studies, differences between the effect of the lin-SB056-1/EDTA combination and the sum of the effects of its individual components (i.e., lin-SB056-1 and EDTA) were analyzed using a Student’s *t*-test for independent samples. A *p*-value < 0.05 was considered statistically significant.

## Results

### Bactericidal Activity of lin-SB056-1 in Combination With EDTA in Host Cell Culture Medium

**FIGURE 1:**
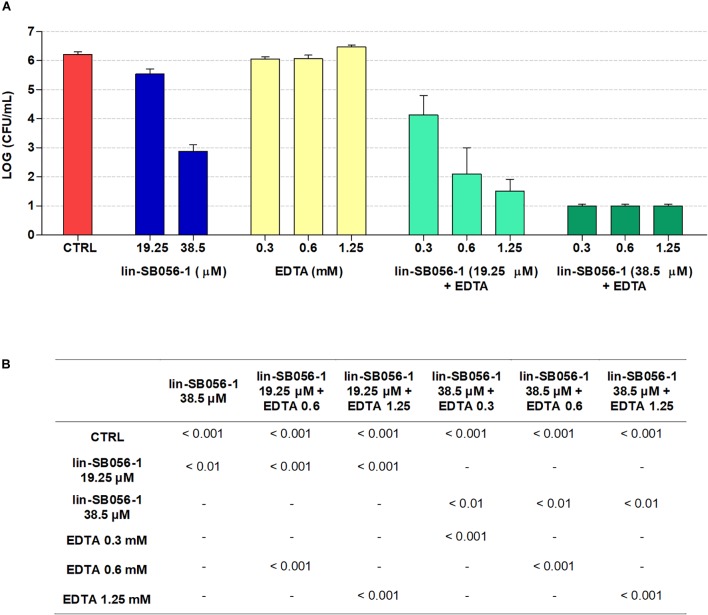
Bactericidal activity of lin-SB056-1, alone and in combination with EDTA, against planktonic cells of *Pseudomonas aeruginosa* PAO1 in GTSF-2 medium after 1.5 h of incubation. **(A)** Bactericidal activity of the lin-SB056-1/EDTA combinations was assessed by CFU count. Control (CTRL) represents untreated bacteria. A number of 10 CFU/mL was taken as detection limit. Data are reported as mean ± standard error of at least three independent experiments. **(B)** *p*-Values obtained by one-way ANOVA followed by Bonferroni *post hoc* test; only statistically significant comparisons are reported.

It is known that culture media can strongly affect the biological properties of AMPs (Dorschner et al., 2006; Ersoy et al., 2017). Therefore, the bactericidal activity of lin-SB056-1, alone and in combination with EDTA, was evaluated in the host cell culture medium (i.e., GTSF-2 medium) in order to identify the most promising peptide/EDTA combinations to be included in the subsequent antibiofilm activity studies. The antibacterial activity against planktonic *P. aeruginosa* PAO1 was assessed after 1.5 h of incubation taking into account the rapid killing kinetics of lin-SB056-1 (Maisetta et al., 2017). The peptide alone was able to reduce the number of viable bacteria by 3-Log units at a concentration of 38.5 μM (Figure 1A). In addition, a significantly increased bactericidal activity was observed when the peptide was used in combination with sub-inhibitory concentrations of EDTA (Figure 1B). In particular, the combination of lin-SB056-1 at 38.5 μM with EDTA (0.3 to 1.25 mM) resulted in the reduction of the initial bacterial inoculum to the limit of detection (10 CFU/mL) (Figure 1A). A statistically significant difference was also obtained by comparing the Log reduction of the lin-SB056-1/EDTA combination with the sum of the Log reductions caused by the individual compounds (data not shown).

### Cytotoxicity of lin-SB056-1 in Combination With EDTA in the 3-D Lung Epithelial Cell Model

**FIGURE 2:**
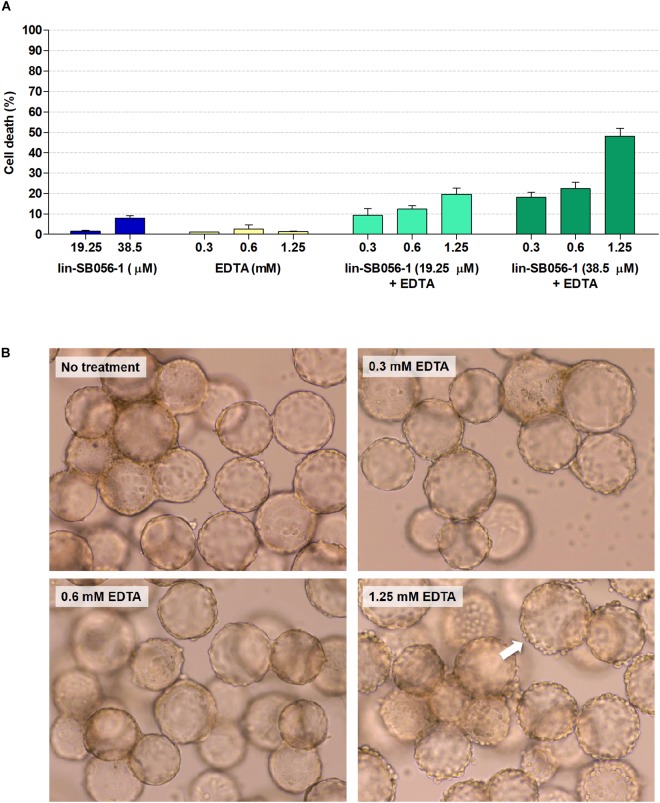
Cytotoxicity of lin-SB056-1 and EDTA, used alone and in combination, toward 3-D lung epithelial cells after 4 h of incubation. **(A)** Cell viability was evaluated by LDH-release assay. Control (CTRL) represents untreated 3-D cells. Data are reported as mean ± standard error of at least three independent experiments. **(B)** Effect of EDTA on cell adhesion to microcarrier bead scaffolds was evaluated by optical microscopy. Images were obtained at a magnification of 1200×. White arrow indicates round-shaped cells detaching from the surface of microcarrier beads.

The effect of lin-SB056-1, used alone and in combination with EDTA, on host cell viability was evaluated in the 3-D lung epithelial cell model. Lin-SB056-1 alone did not significantly affect host cell viability at any of the tested concentrations, as determined through the measurement of LDH release (Figure 2A). Similarly, no cytotoxic effect was observed when EDTA was used alone in the concentration range 0.3–1.25 mM. When used in combination with EDTA, the peptide exerted enhanced cytotoxicity toward 3-D cells although the fraction of dead cells was below 22% for all tested peptide/EDTA combinations, with the exception of the combination of 38.5 μM lin-SB056-1 and 1.25 mM EDTA (approximately 50% cell death) (Figure 2A). Furthermore, due to its chelating properties, the effect of EDTA on host cell adhesion was assessed through the microscopic observation of 3-D aggregates. Although EDTA was non-toxic when used alone (Figure 2A), at the concentration of 1.25 mM, it caused a morphological change of 3-D structures with the appearance of round-shaped cells and noticeable cell detachment from the scaffolds (Figure 2B). Nevertheless, morphology of 3-D cells treated with lower concentrations of EDTA (0.3 and 0.6 mM) was similar to the untreated control (Figure 2B), even when used in combination with the peptide (data not shown). Accordingly, subsequent biofilm studies were performed by selecting peptide/EDTA combinations able to reduce the number of planktonic bacteria by at least 3-Log units with as low as possible effect on cell viability (less than 22% cell death) and adhesion (i.e., lin-SB056-1 at 19.25 and 38.5 μM combined with EDTA at 0.3 and/or 0.6 mM).

### Biofilm-Inhibitory Activity of lin-SB056-1 in Combination With EDTA in the 3-D Lung Epithelial Cell Model

**FIGURE 3:**
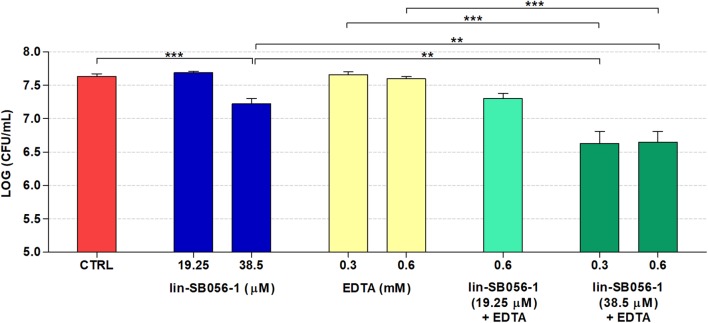
Biofilm-inhibitory activity of lin-SB056-1, alone and in combination with EDTA, against *P. aeruginosa* PAO1 in the 3-D lung epithelial cell model after 4 h of incubation. Control (CTRL) represents untreated bacteria. Data are reported as mean ± standard error of at least three independent experiments. ^∗∗^*p* < 0.01; ^∗∗∗^*p* < 0.001 (Kruskal–Wallis test followed by Bonferroni *post hoc* test).

The peptide/EDTA combinations selected according to bactericidal activity and cytotoxicity studies were tested for their ability to inhibit biofilm formation by *P. aeruginosa* PAO1 in the 3-D lung epithelial cell model after 4 h of incubation. Incubation time for the evaluation of the antibiofilm activity was selected taking into account that *P. aeruginosa* PAO1 is able to form microcolonies on the 3-D lung epithelium starting from 2 h of incubation (Crabbé et al., 2017). When assayed alone, lin-SB056-1 (38.5 μM) resulted in a decrease of approximately 0.5 Log-unit (3.3 fold) in the number of biofilm-forming bacteria compared to the untreated control (Figure 3). Among all the tested combinations, the most powerful biofilm-inhibitory effect in terms of viable count reduction (approximately 1 Log [10 fold] as compared to the control) was obtained by combining lin-SB056-1 at 38.5 μM with EDTA at 0.3 and 0.6 mM (Figure 3). At such concentrations, a statistically significant reduction in the CFU number was observed between the combination and the peptide or EDTA used alone (Figure 3). A statistically significant difference (*p* < 0.05, independent samples *t*-test) was also obtained by comparing the Log reduction of the lin-SB056-1/EDTA combination with the sum of the Log reductions caused by the individual compounds.

### Activity of (lin-SB056-1)_2_-K in the 3-D Lung Epithelial Cell Model

**FIGURE 4:**
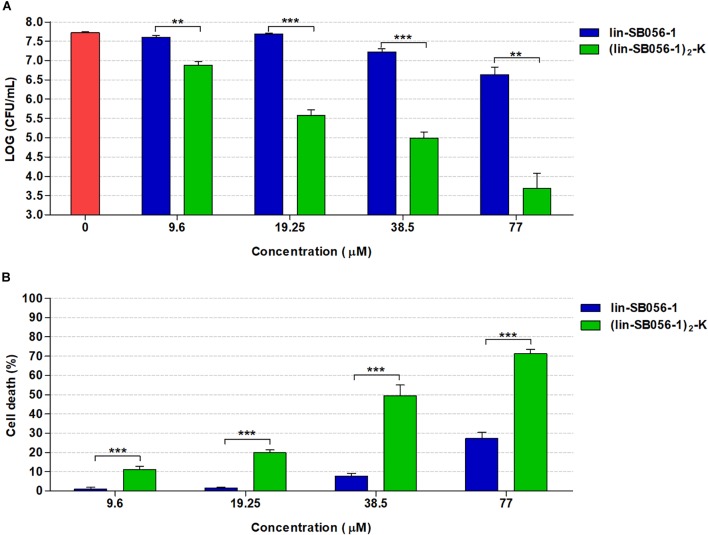
Comparison of the biofilm-inhibitory activity and cytotoxicity of lin-SB056-1 and (lin-SB056-1)_2_-K in the 3-D lung epithelial cell model after 4 h of incubation. **(A)** Biofilm-inhibitory activity against *P. aeruginosa* PAO1 was assessed by CFU count. Data are reported as mean ± standard error of at least three independent experiments. ^∗∗^*p* < 0.01; ^∗∗∗^*p* < 0.001 (Kruskal–Wallis test followed by Bonferroni *post hoc* test). **(B)** Viability of 3-D lung epithelial cells was evaluated by LDH-release assay. Data are reported as mean ± standard error of at least three independent experiments. ^∗∗∗^*p* < 0.001 (one-way ANOVA followed by Bonferroni *post hoc* test).

The biofilm-inhibitory activity and cytotoxicity of lin-SB056-1 were compared to those exerted by its dendrimeric derivative (lin-SB056-1)_2_-K in order to explore the possibility to obtain an improved antibiofilm effect in the 3-D lung epithelial cell model. The antibacterial activity of the two peptides was preliminarily compared in terms of MIC values, evaluated in MHB and GTSF-2 medium. Interestingly, although (lin-SB056-1)_2_-K was found to exhibit 2 to 4-fold higher MIC values in MHB than lin-SB056-1 against *P. aeruginosa* PAO1 and CF lung isolates (AA2 and AA44), it exerted an enhanced antibacterial activity in GTSF-2 medium (Table 1). Accordingly, (lin-SB056-1)_2_-K demonstrated a significantly improved ability to inhibit biofilm formation by *P. aeruginosa* PAO1 as compared to its linear counterpart in the 3-D lung epithelial cell model (Figure 4A). Although dendrimerization of the peptide was associated with an overall increase in cytotoxicity toward 3-D A549 cells (Figure 4B), (lin-SB056-1)_2_-K at a concentration causing less than 20% cell death (19.25 μM) (Figure 4B) reduced the number of biofilm-forming bacteria by 2-Log units (100 fold) (Figure 4A). Thus, when compared at the same cut-off of cytotoxicity (20% cell death) (Figure 4B), the dendrimeric form of the peptide (at 19.25 μM) caused an additional 1.5-Log unit reduction in the CFU count as compared to its linear counterpart (at 38.5 μM) (Figure 4A).

**Table 1:** Minimal inhibitory concentration (MIC) values of lin-SB056-1 and (lin-SB056-1)_2_-K against *P. aeruginosa* PAO1 and CF clinical isolates evaluated in MHB and GTSF-2 medium.

	lin-SB056-1		(lin-SB056-1)_2_-K	
		
	MHB	GTSF-2	MHB	GTSF-2
PAO1	4.8^a^	38.5	19.25	9.6
AA2	9.6	77	38.5	9.6
AA44	4.8	38.5	9.6	2.4


*^*a*^Concentrations are expressed in μM.*

Higher levels of cytotoxicity of the dendrimeric peptide over the monomeric counterpart were also observed in a standard hemolysis assay. Nevertheless, at concentrations exerting significant antibiofilm activity (up to 19.25 μM), (lin-SB056-1)_2_-K caused less than 5% hemolysis (Supplementary Figure S1).

**FIGURE 5:**
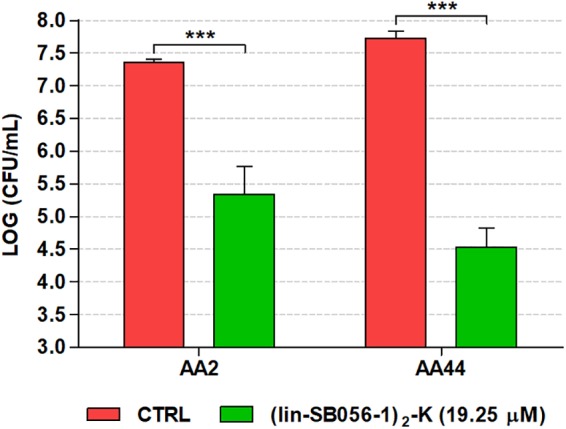
Biofilm-inhibitory activity of (lin-SB056-1)_2_-K (19.25 μM) against CF isolates of *P. aeruginosa* in the 3-D lung epithelial cell model after 4 h of incubation. Control (CTRL) represents untreated bacteria. Data are reported as mean ± standard error of at least three independent experiments. ^∗∗∗^*p* < 0.001 (independent samples *t*-test or Mann–Whitney test).

Importantly, (lin-SB056-1)_2_-K also exhibited a marked ability to inhibit biofilm formation by an early (AA2) and a late (AA44) clinical isolate of *P. aeruginosa* from a CF patient. In particular, at the concentration of 19.25 μM, the peptide reduced the number of CFU up to 3-Log units (1000 fold) as compared to the untreated control (Figure 5).

**FIGURE 6:**
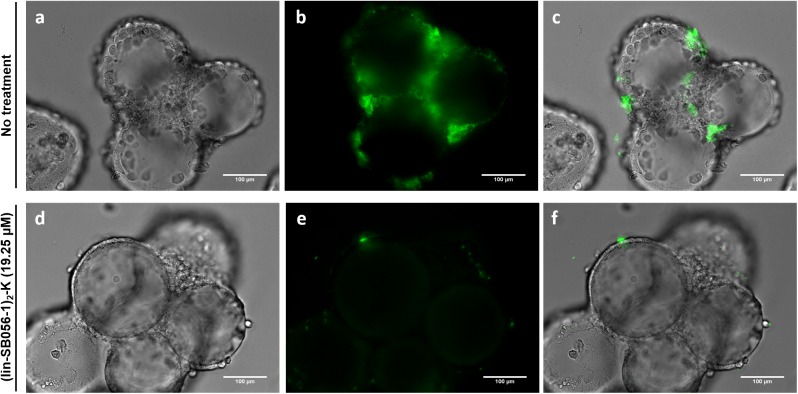
Qualitative evaluation of the biofilm-inhibitory activity of (lin-SB056-1)_2_-K (19.25 μM) against GFP-expressing *P. aeruginosa* PAO1 in the 3-D lung epithelial cell model after 4 h of incubation. Light microscopy **(a,d)**, epifluorescence **(b,e)**, and overlaid images **(c,f)** were obtained at a magnification of 599×. Scale bar corresponds to 100 μm.

Qualitative assessment of the antibiofilm activity of (lin-SB056-1)_2_-K in the 3-D lung epithelial cell model was performed by monitoring the formation of fluorescent biofilm-like structures by GFP-producing *P. aeruginosa* PAO1. A remarkable decrease in the amount of biofilm-like structures associated with 3-D lung epithelial cells was observed after the treatment with (lin-SB056-1)_2_-K at 19.25 μM (Figure 6).

### Biofilm-Inhibitory Activity of (lin-SB056-1)_2_-K in Artificial Wound Model

**FIGURE 7:**
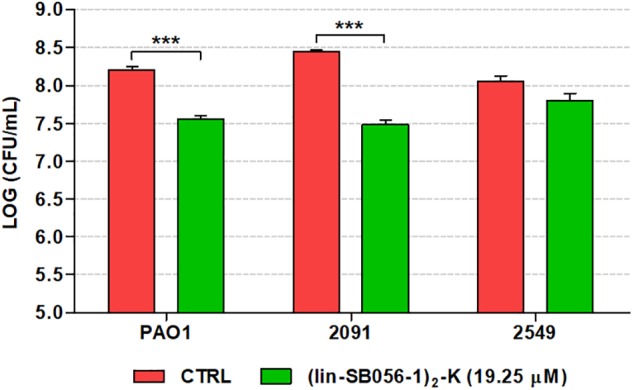
Biofilm-inhibitory activity of (lin-SB056-1)_2_-K (19.25 μM) against *P. aeruginosa* PAO1 and wound clinical isolates in the *in vitro* artificial wound model after 16 h of incubation. Control (CTRL) represents untreated bacteria. Data are reported as mean ± standard error of at least three independent experiments. ^∗∗∗^*p* < 0.001 (independent samples *t*-test).

The efficacy of (lin-SB056-1)_2_-K against forming biofilms of *P. aeruginosa* was also investigated in an *in vitro* chronic wound model consisting of a dermis-like scaffold embedded in physiological levels of blood components. At 19.25 μM, (lin-SB056-1)_2_-K displayed a significant biofilm-inhibitory activity against both *P. aeruginosa* PAO1 and the wound clinical isolate 2091, reducing the number of viable bacteria up to 1 Log-unit (10 fold) after 16 h of incubation (Figure 7). Although the peptide resulted in a reduction of approximately twofold in the CFU count of the clinical strain 2549, the difference did not reach statistical significance as compared to the control (*p* = 0.058, independent samples *t*-test).

## Discussion

Over the last years, an increasing awareness of the role of biofilms in the establishment of chronic infections has led to a growing interest in the identification and development of novel antibiofilm strategies. High tolerance of biofilms to available antibiotics has stimulated interest in AMPs as potential antibiofilm agents (Batoni et al., 2011; Di Luca et al., 2015; de la Fuente-Núñez et al., 2016). Nevertheless, most of the studies assessing the antibiofilm activity of candidate AMPs have been performed using *in vitro* models in which the conditions are very different from those found *in vivo* at the site of infection (Lebeaux et al., 2013; Roberts et al., 2015). In particular, the electrostatic interaction of AMPs with bacterial membranes can be dramatically reduced by the presence of salts at physiological levels, proteases, macromolecules of the body fluids as well as host cells (Dorschner et al., 2006; Hancock and Sahl, 2006; Maisetta et al., 2008). Furthermore, many of these *in vitro* models rely on the formation of surface-attached biofilms characterized by a biomass order of magnitude larger than *in vivo* biofilms. Indeed, it has been reported that *in vitro* biofilms can reach dimensions of approximately 1 to 10 cm^2^ with a thickness up to 300 μm, whereas biofilm aggregates in chronic infections have been shown to mainly range from 5 to 200 μm in diameter (Bjarnsholt et al., 2013). In addition, different gene expression patterns and susceptibility to most antimicrobials have been reported for *in vitro* and *in vivo*-like biofilms (Caceres et al., 2014; Crabbé et al., 2017). For example, *P. aeruginosa* biofilms formed on CF bronchial epithelial cells have been found to exhibit enhanced tolerance to tobramycin as compared to biofilms grown on plastic surfaces (Anderson et al., 2008; Moreau-Marquis et al., 2008). Therefore, conclusions drawn from standard laboratory techniques may not be directly translated to human infections, and physiologically relevant biofilm model systems are desirable as a complementary tool for the evaluation of novel AMP-based antimicrobial agents (Pompilio et al., 2015; Bock et al., 2018; Dostert et al., 2018).

To address this issue, we exploited an *in vivo*-like 3-D lung epithelial cell model and an artificial wound model in order to assess the therapeutic potential of the semi-synthetic peptide lin-SB056-1 and its dendrimeric derivative (lin-SB056-1)_2_-K in the context of *P. aeruginosa* biofilm infections. Besides mimicking key aspects of the lung tissue, *P. aeruginosa* has been previously reported to form biofilms in the 3-D lung epithelial cell model (Crabbé et al., 2017), which is relevant for the phenotype of this opportunistic pathogen found during chronic lung infections (Alhede et al., 2011; Bjarnsholt et al., 2013; Ciofu et al., 2015). Additionally, the presence of host cells has been shown to markedly influence the ability of different antimicrobial agents to inhibit the formation of *P. aeruginosa* biofilms. Significant differences in the efficacy of conventional antibiotics (amikacin, colistin, gentamicin, and tobramycin) and the antibiofilm peptide DJK-5 have been observed between biofilms associated with 3-D lung epithelial cells and biofilms formed on plastic surfaces (Crabbé et al., 2017). Interestingly, the 3-D lung epithelial cell model offers the possibility to assess in parallel the antibiofilm activity and the cytotoxicity of antimicrobials toward cell types representative of the site of infection. In the case of AMPs, the tendency to employ hemolytic activity as preferred parameter for the high-throughput screening of cytotoxicity has markedly limited the accurate evaluation of their potential adverse effects. Indeed, the degree to which AMPs permeabilize human erythrocytes may not realistically reflect their selective toxicity against specific cell types in complex biomatrices *in vivo* (Yeaman and Yount, 2003; Bacalum and Radu, 2015). In addition to the lung environment, we aimed at recreating the physiological conditions encountered in chronic wounds by employing an artificial dermis embedded in a blood-based medium (Sun et al., 2008; Brackman et al., 2016). Despite the absence of a biotic surface, the artificial wound model closely reflects *in vivo* wound beds by resembling the nutritional conditions found in wound exudates and the dermal surface on which bacteria can adhere and form biofilm-like structures (Brackman et al., 2016; Trivedi et al., 2017).

In a previous study, we have demonstrated the effectiveness of the co-treatment with lin-SB056-1 and EDTA in preventing *P. aeruginosa* biofilms in an abiotic environment similar to the CF airway (Maisetta et al., 2017). Combination treatments represent a promising strategy to enhance the efficacy of AMPs against bacterial biofilms, especially under *in vivo*-like conditions that are known to interfere with peptide activity (Walkenhorst, 2016; Grassi et al., 2017b). In particular, the use of the chelating agent EDTA has been shown to improve the activity of different peptides due to its disaggregating effect on the biofilm extracellular matrix and its perturbing action on the outer membrane of Gram-negative bacteria (Banin et al., 2006; Grassi et al., 2017c). Furthermore, the ability of EDTA to decrease mucus viscosity has been hypothesized to promote the mobility and killing activity of lin-SB056-1 in the presence of artificial sputum (Gustafsson et al., 2012; Maisetta et al., 2017). In the present study, the synergistic interaction between lin-SB056-1 and EDTA has also been confirmed in the 3-D lung epithelial cell model. Indeed, lin-SB056-1 in combination with sub-inhibitory concentrations of EDTA significantly reduced the formation of *P. aeruginosa* biofilms in association with the airway epithelium.

In order to further improve the therapeutic potential of lin-SB056-1, we investigated the antibiofilm properties of the peptide’s dimeric derivative (lin-SB056-1)_2_-K in the same 3-D lung infection model. Interestingly, (lin-SB056-1)_2_-K was remarkably more effective than its monomeric counterpart in preventing the formation of *P. aeruginosa* biofilm-like structures in association with 3-D lung epithelial cells. Although in agreement with peptides’ activity observed in GTSF-2, these results contrast with MIC values obtained in MHB, where (lin-SB056-1)_2_-K consistently exhibited MIC values higher than lin-SB056-1 against both PAO1 strain and CF lung isolates of *P. aeruginosa*. This observation indicates that medium composition may greatly affect peptide activity, suggesting that the widely used MHB might not be an appropriate medium to screen the antibacterial potency of candidate AMPs (Dostert et al., 2018). The significant difference between the MIC values obtained in MHB and GTSF-2 could be due to differential peptide binding to specific medium components, such as complex carbohydrates (e.g., starch) that are present in MHB but not in GTSF-2, and/or to proteins (Lelkes et al., 1997). Different amounts of cations and carbonate in these media could also influence peptide activity in a manner that is difficult to forecast (Dorschner et al., 2006; Wu et al., 2008).

It has been suggested that the concentration at which a peptide exerts its antibiofilm activity is a parameter that could provide valuable information about its mechanism of action (Batoni et al., 2016a; Pletzer et al., 2016). Under the test conditions, both lin-SB056-1 and (lin-SB056-1)_2_-K inhibited adhesion/biofilm formation in association with host cells at concentrations equal and/or higher than their MIC values in GTSF-2 medium, thereby suggesting that they may act by a “classical” antibacterial effect rather than via a biofilm-specific mechanism of action.

As expected, along with the enhancement of the biofilm-inhibitory activity, dimerization of lin-SB056-1 led to an overall increase in the cytotoxicity of the original peptide against both 3-D lung epithelial cells and human erythrocytes. An increase in peptide cytotoxicity following modification is not surprising as it is widely accepted that there is a direct relationship between the antimicrobial and the cytotoxic properties of AMPs (Matsuzaki, 2009; Takahashi et al., 2010; Bolt et al., 2017). Nevertheless, in the 3-D lung epithelial cell model, a net percentage of cell death lower than 20% was observed at concentrations of (lin-SB056-1)_2_-K that significantly inhibited biofilm formation (up to 1000-fold reduction in the number of biofilm-forming bacteria) by both the reference strain PAO1 and two CF clinical isolates of *P. aeruginosa.* Interestingly, when evaluated at the same cut-off of cytotoxicity (20% cell death), (lin-SB056-1)_2_-K resulted in a superior antibiofilm activity also compared to lin-SB056-1 used in combination with EDTA, causing an additional reduction of 1 to 1.5-Log units in the CFU number as compared to the selected lin-SB056-1/EDTA combinations (i.e., 19.25 μM lin-SB056-1 in combination with 0.6 mM EDTA, and 38.5 μM lin-SB056-1 in combination with 0.3 mM EDTA).

Overall, our findings underline the superiority of the dendrimeric arrangement over the monomeric structure for the maintenance of the antimicrobial activity in complex and physiologically relevant environments. Interestingly, the biofilm-inhibitory activity of (lin-SB056-1)_2_-K was also maintained against different *P. aeruginosa* strains in the artificial wound model under very challenging *in vivo*-like conditions due to the presence of high concentrations of plasma/blood, which are known to impact the activity of peptides (Maisetta et al., 2008). It is commonly recognized that dendrimeric peptides exhibit enhanced antimicrobial activity as compared to their monomeric counterparts due to the higher local concentration of bioactive units and the reduced susceptibility to proteolytic degradation (Lind et al., 2015; Pires et al., 2015). In addition, it is likely that (lin-SB056-1)_2_-K is less influenced than lin-SB056-1 by the presence of physiological salt concentrations, as reported for the original dimeric structure from which it was derived (i.e., den-SB056-1) (Batoni et al., 2016a).

Although (lin-SB056-1)_2_-K at the active concentrations exhibited a certain degree of cytotoxicity against both erythrocytes and lung epithelial cells, different strategies could be exploited in the attempt to reduce peptide adverse effects against mammalian cells, while maintaining its antimicrobial properties. A number of different approaches have been described to control cell selectivity of AMPs, including modulation of a range of physicochemical parameters (e.g., hydrophobicity, helicity, and amphipathicity), design of pro-peptides, and insertion of D-amino acids and/or non-natural residues in the peptide primary sequence (Matsuzaki, 2009; Takahashi et al., 2010; Forde et al., 2016). Among these strategies, peptide entrapment in suitable nanocarriers able to deliver the active molecules to the infection site, while increasing their stability and minimizing side effects, appears one of the most promising approaches, and is currently under evaluation in our laboratory (Piras et al., 2015; Sandreschi et al., 2016).

## Conclusion

In order to narrow the gap between *in vitro* and *in vivo* observations, research on AMPs should take into account the influence of the host environment on biofilm phenotype and peptide activity. Several studies have extensively questioned the usefulness of currently performed antibiotic susceptibility testing due to their limited clinical predictive value (Hurley et al., 2012; Hancock, 2015; Coenye et al., 2018). Analysis of antimicrobial activity in host-mimicking conditions represents a convenient approach to make a more realistic prediction of the therapeutic success of AMPs, thereby avoiding testing unpromising peptides in costly animal studies and clinical trials (Dorschner et al., 2006; Dostert et al., 2018). Although clinical development of novel drugs requires evidence of *in vivo* efficacy in animal models, preliminary *in vitro* evaluation in physiologically relevant biofilm model systems may facilitate the identification of effective AMP-based antimicrobials and reduce animal testing. For instance, the ability of (lin-SB056-1)_2_-K to preserve a significant antibiofilm activity in different challenging host-mimicking environments suggested that such peptide might be a promising template for the development of novel treatment strategies against *P. aeruginosa* infections.

## Author Contributions

AC, GB, LG, and TC conceived, designed, and drafted the study. LG, LO, PR, and SVdB contributed to the acquisition, analyzed and interpreted the data. AC, AR, GB, GM, LG, LO, PR, SE, SVdB, and TC critically revised the study and gave the final approval.

## Conflict of Interest Statement

The authors declare that the research was conducted in the absence of any commercial or financial relationships that could be construed as a potential conflict of interest.

## Footnotes

1 www.baamps.it

## Abbreviations

AMP: antimicrobial peptide
BB: Bolton broth
CF: cystic fibrosis
CFUs: colony-forming units
Col: collagen
EDTA: ethylenediaminetetraacetic acid
FBS: fetal bovine serum
HA: hyaluronic acid
HBSS: Hanks’ balanced salt solution
hRBCs: human red blood cells
LB: Luria-Bertani broth
LDH: lactate dehydrogenase
MHB: Mueller-Hinton broth
MIC: minimal inhibitory concentration
PSS: physiological saline solution
RWV: rotating-wall vessel
TSA: tryptone soy agar
TSB: tryptone soy broth
WLM: wound-like medium
