# Supplementary figures and images for: The Antimicrobial Peptide lin-SB056-1 and Its Dendrimeric Derivative Prevent Pseudomonas aeruginosa Biofilm Formation in Physiologically Relevant Models of Chronic Infections
Source: Front Microbiol. 2019 Feb 8;10:198. doi: 10.3389/fmicb.2019.00198 (PMC6376900; doi:10.3389/fmicb.2019.00198)

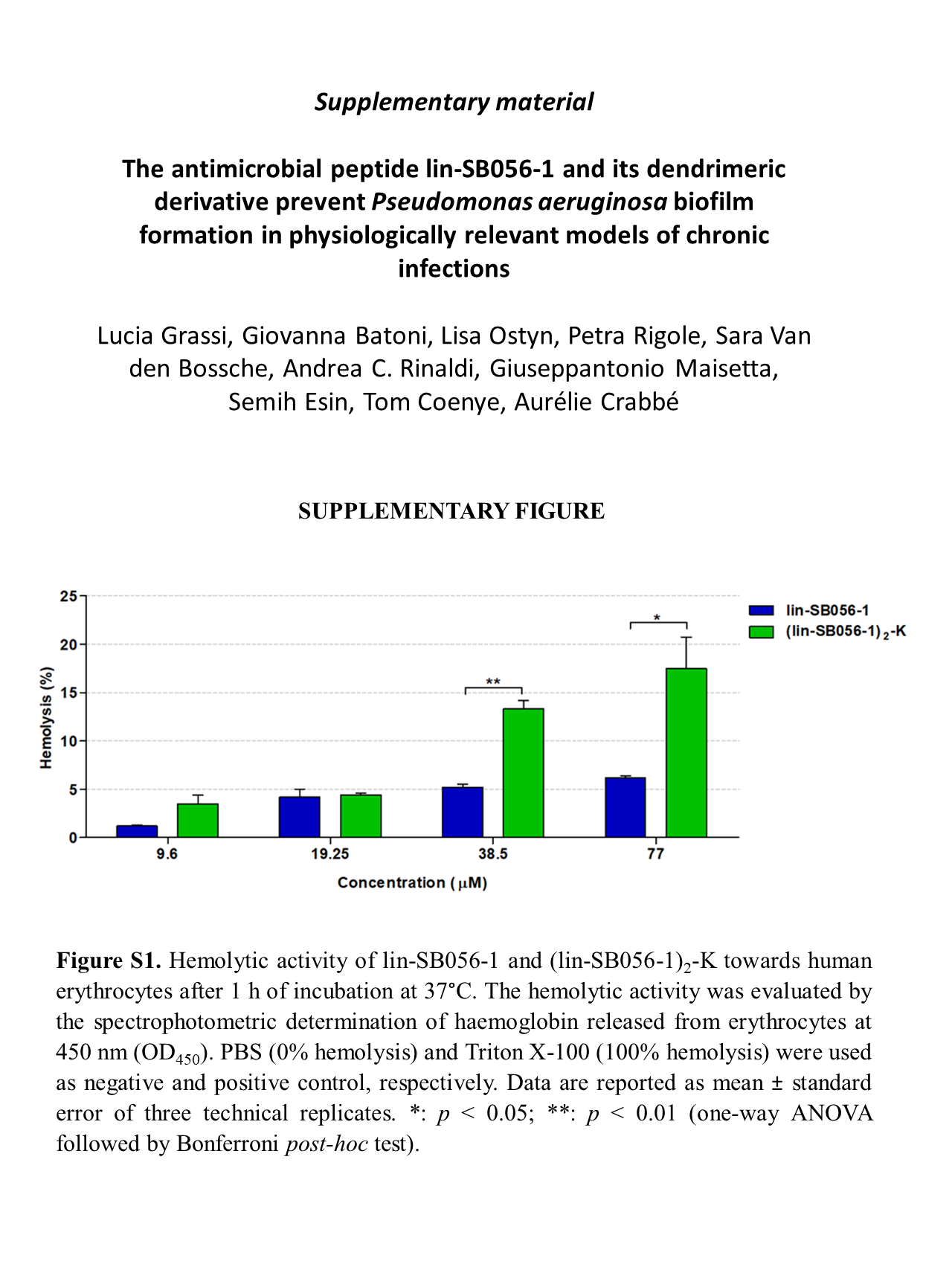

Supplement: Supplementary file 1 [file Image_1.TIF]
